# Global Picture of Genetic Relatedness and the Evolution of Humankind

**DOI:** 10.3390/biology9110392

**Published:** 2020-11-10

**Authors:** Gennady V. Khvorykh, Oleh A. Mulyar, Larisa Fedorova, Andrey V. Khrunin, Svetlana A. Limborska, Alexei Fedorov

**Affiliations:** 1 Institute of Molecular Genetics of National Research Centre Kurchatov Institute, Moscow 123182, Russia; khvorykh@img.ras.ru (G.V.K.); khrunin@img.ras.ru (A.V.K.); limbor@img.ras.ru (S.A.L.); 2 CRI Genetics LLC, Santa Monica, CA 90404, USA; amulyar@crigenetics.com (O.A.M.); lvfedorova3@gmail.com (L.F.); 3 Department of Medicine, University of Toledo, Toledo, OH 43614, USA

**Keywords:** genealogy, computational biology, genomics, inheritance, polymorphism, genetic variation

## Abstract

**Simple Summary:**

The intricacies of human ancestry are buried deep within our DNA. For years, scientists have been working to piece together a vast picture of our genetic lineage. The purpose of this study was to further reveal this global picture of human genetic relatedness using identical-by-descent (IBD) genomic fragments. We processed over 65 million very rare single nucleotide polymorphic (SNP) alleles and detected over 17 million shared IBD fragments, including very short IBD fragments that allowed us to trace common ancestors back to 200,000 years ago. We also determined nine geographical regions representing nine unique genetic components for mankind: East and West Africa, Northern Europe, Arctica, East Asia, Oceania, South Asia, Middle East, and South America. The levels of admixture in every studied population could be assigned to one of these regions and long-term neighboring populations are strikingly similar, despite any political, religious, and cultural differences. Additionally, we observed the topmost admixture to be in central Eurasia. The entire picture of relatedness of all the studied populations presents itself in the form of shared number/size of IBDs, providing novel insights into geographical admixtures and genetic contributions that shaped human ancestry into what it is today.

**Abstract:**

We performed an exhaustive pairwise comparison of whole-genome sequences of 3120 individuals, representing 232 populations from all continents and seven prehistoric people including archaic and modern humans. In order to reveal an intricate picture of worldwide human genetic relatedness, 65 million very rare single nucleotide polymorphic (SNP) alleles have been bioinformatically processed. The number and size of shared identical-by-descent (IBD) genomic fragments for every pair of 3127 individuals have been revealed. Over 17 million shared IBD fragments have been described. Our approach allowed detection of very short IBD fragments (<20 kb) that trace common ancestors who lived up to 200,000 years ago. We detected nine distinct geographical regions within which individuals had strong genetic relatedness, but with negligible relatedness between the populations of these regions. The regions, comprising nine unique genetic components for mankind, are the following: East and West Africa, Northern Europe, Arctica, East Asia, Oceania, South Asia, Middle East, and South America. The level of admixture in every studied population has been apportioned among these nine genetic components. Genetically, long-term neighboring populations are strikingly similar to each other in spite of any political, religious, and cultural differences. The topmost admixture has been observed at the center of Eurasia. These admixed populations (including Uyghurs, Azerbaijanis, Uzbeks, and Iranians) have roughly equal genetic contributions from the Middle East, Europe, China, and India, with additional significant traces from Africa and Arctic. The entire picture of relatedness of all the studied populations unfolds and presents itself in the form of shared number/size of IBDs.

## 1. Introduction

The global picture of human genetic relatedness still includes many controversial aspects. This controversy stems mainly from the widespread dissemination of human populations across the world due to various migrations, trading, and conquest events that occurred multiple times over thousands of years. In addition, the intricate nature of DNA inheritance, such as extensive meiotic recombination, obscures our genetic studies, hindering development of complete genetic histories. Nevertheless, certain methods may allow us to overcome these technical impediments. Every human has two parents, four grandparents, eight great-grandparents, and so on, doubling the direct number of ancestors every generation. Following this pattern, a person should have over a thousand direct genealogical ancestors at the tenth generation, over a million at the 20th generation, over a billion at the 30th, and over a trillion at the 40th. However, the entire population size of our 40th generation ancestors, who lived around one thousand years ago, is well below a billion. Clearly many of those 1000-year-old genealogical ancestors had multiple genealogical pathways of relatedness to every person within the current population, as well as to all other people from the same region. On another note, over 99.9% of direct genealogical 40th-generation ancestors for a given individual have not passed on any fragments of their own genomic DNA to this person. This is because those chromosomal fragments were randomly lost somewhere along the 40th generation genealogy tree [1]. Only a tiny fraction of these direct ancestors passed fragments of their chromosomes to the individual under consideration. In the vast majority of cases, furthermore, each “lucky” ancestor only passes on one piece of DNA that, on average, is 2.1 million nucleotides long. For context, this is about the size of a bacterial genome, and represents just 0.064% of the entire human genome. However, due to the stochastic nature of DNA recombination and sexual transmission, some of these inherited DNA segments may be many times shorter or longer than average. These pieces of DNA, that have been transmitted from ancestor to progenitor, are known as identical-by-descent (IBD) fragments. IBD fragments can be distinguished from analogous pieces of DNA by the unique patterns created by numerous mutations inside each fragment. Currently, we are aware of about 700 million single nucleotide polymorphic (SNP) sites in the human genome [2]. For any long chromosomal fragment (that say contains 1 million nucleotides), there should be a sufficient number of polymorphic sites that distinguish it from other DNA fragments located in the same genomic region in other individuals. This observation suggests that detecting IBD fragments in the human genome back to the nth generation is fairly straightforward. However, the average size of IBD fragments is inversely proportional to the number of passing generations. Thus, our ability to detect IBD fragments has had an observed limit at ~200,000 years in the past. Specifically, shared IBD fragments for the last common ancestor from ~200,000 years ago frequently become smaller than 10,000 nucleotides. Even evaluating genetic relatedness, when the last common ancestor lived several thousand years ago, requires accumulation of good statistics that may only be obtained from analyzing multiple individuals with the genomes sequenced with quite high quality.

The exploration of genetic relatedness is based on the identification of shared IBD fragments between groups of people. IBDs are identified using various statistical algorithms that analyze the patterns of single nucleotide polymorphisms (SNPs). Most of these algorithms are mathematically sophisticated and operate with abstract statistical values and parameters that do not have obvious biological correlates. For example, genetic relatedness may be measured by Multiple Sequentially Markovian Coalescent (MSMC) statistics [3,4], putative ghost haplotypes (PGH) [5], or Principal Component Relate statistics [6], among others. In contrast to these statistics, we developed a relatively simple computational technique for the direct identification of shared IBD fragments between groups of people [1]. Our approach characterizes clusters (five or more) of very rare neighboring SNP alleles that unambiguously serve as markers for shared IBD fragments. This method allows us to identify tiny IBD fragments that may be as short as 10,000 nucleotides in length. One of the main advantages of our approach is that it does not require “phasing” of SNP datasets; e.g., statistical separation of sequenced diploid genomes into parent-1 and parent-2 haploid counterparts, which creates occasional but unavoidable mistakes. Additionally, our computations are easily comprehensible for the general public and enable future detailed investigation of any individual IBD fragments and their inheritance pathways.

There have been several successful, large-scale projects for sequencing multiple human genomes, with inference of population relatedness. One of the first pivotal projects was the 1000 Genomes Project, which characterized 2500 people from 26 populations [7]. Afterwards, there was the Simons Genome Diversity Project (SGDP), which characterized 300 genomes from 142 populations [8]; the Estonian Biocentre Human Genome Diversity Panel (EGDP) with 379 new genomes from 125 populations [4]; and Human Genome Diversity Panel HGDP-CEPH [9], among others. There is an ongoing project for characterization of 100,000 people across Asia [10]. Also, there have been several projects that specifically characterized particular populations in high detail. These include: The characterization of 128 Ashkenazi Jewish genomes from New York [11], 100,000 genomes from England [12], 27,566 Icelandic genomes [13], and 2500 genomes from different Russian ethnic groups (The Genome Russia Project [14]). In addition to modern people, several genomes of Archaic lineages and several thousand genomes of Ancient people who lived from several hundred to a thousand years ago have been characterized [15].

The main goal of the study was to reveal a global picture of genetic relatedness and the evolution of mankind. Combining SNP from several milestone whole-genome sequencing datasets, that present a vast spectrum of populations across all continents as well as archaic and ancient people, became an important task. One of the major challenges we faced was associated with the differing qualities of sequencing within different datasets. Besides significant variations in sequencing coverage, various scientific groups used different computational parameters for SNP characterization, and this resulted in large variations in the number of alternative SNP alleles in the individual genomes. We demonstrate here that all these technical predicaments are solvable. We present an extensive dataset of 17 million shared IBD chromosomal fragments, which resulted from pairwise comparison of 3127 individual genomes from 232 populations and seven prehistoric people, all obtained from public datasets. A crucial aspect of our project is the comparison between a broad spectrum of human populations from all major groups around the world and prehistoric people. Each were assessed with the same rules and statistics. Our earliest detected relatedness event in pre-human history was the admixture of Neanderthals with African populations that occurred approximately 200,000 years ago, potentially even earlier. We also observed admixture of African and Oceania populations around 125,000 years ago. The results should be of great interest to anthropologists, historians, linguists, evolutionists, and those interested in the origins and migrations of humans.

## 2. Results

### 2.1. IBD Statistics

Shared IBD chromosomal fragments between a pair of individuals are defined by clusters of neighboring very rare SNP alleles common to two individuals. In this study, we analyzed the whole sequenced genomes of 3121 people from 234 populations, as well as 7 ancient genomes and two simulated genomes described in the Section 5 and Supplementary Table S2. All possible 4,890,628 pairings from these modern and ancient people have been computationally processed. This allowed identification of 17,209,949 shared IBD fragments (Supplementary File S1). To visualize the relationships between individuals, at first, we projected the matrix with the numbers of pairwise shared IBD fragments onto two-dimensional space using multidimensional scaling. The resulting plot is shown in Supplementary Figure S1. Being quite illustrative, the figure does not allow exploring the population relationships in full details. Moreover, the sheer quantity of these data makes it difficult to appreciate them in full detail. Therefore, we integrated the initial data and drew our analyses at the populational level. In other words, ***N*** individuals from population ***A*** have been compared to ***M*** individuals from population ***B***. We analyzed all the possible ***N× M*** pairs that contained one individual belonging to ***A*** and one belonging to ***B***, and from this analysis, we calculated the average number of shared IBD fragments per ***A***/***B*** pair of individuals. This dataset of shared IBD fragments between populations is presented in a 243 × 243 supplementary table (Table S1) as well as in an illustrative fragment (Table 1). Table S1 demonstrates that populations geographically separated from each other (i.e., lived on different continents) frequently share ≤1 IBD fragments per pair, while geographically neighboring populations often share 10–50 IBD fragments per pair.

**Figure 1:**
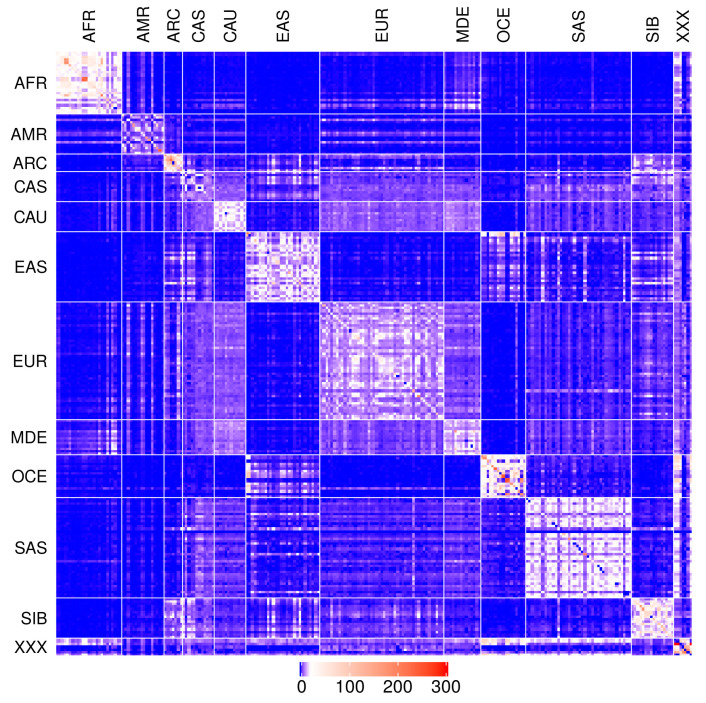
Heat map for the Table S1. Blue color—no genetic relatedness between populations, red—the highest relatedness. The scale represents numbers of IBD fragments per pair of individuals from 0 (dark blue) to 300 (red). AFR—Africa; AMR—America; ARC—Arctic; CAS—Central Asia; CAU—Caucasus; EUR—Europe; EAS—East Asia; SAS—South Asia; OCE—Oceania and Australia; MDE—Middle East; SIB—Siberia; XXX—archaic and prehistoric modern humans described in the Section 5.

Although it is scaled to a populational level, Table S1 is still very large. Thus, to further exhibit the full scope of the information, we also depict it in a heat map (Figure 1). In addition, we ranked every row of data in Table S1 by the number of shared IBD fragments with other populations. An example of this ranking system is presented in Table 2, where the reference population is Russian (Rus_EUR). The Rus_EUR data clearly illustrate the universal trend in our study, that the largest numbers of IBD fragments a population shares are with itself and its close geographic neighbors, which is in accordance with previous observations [16]. In the case of Rus_EUR, Russians share the most IBD fragments with their current internal ethnic groups (Vepsas, Karelians) as well as their historical geographic neighbors such as the Baltic Republics, Finland, Belarus, and Poland (Table 2). In the next tier, Russians share roughly half as many IBD fragments with Western Europeans and other semi-remote neighbors from the south. Finally, the smallest percentages of shared IBD fragments in the Russian population are connected to populations from other continents. The ranking system data for each of the studied populations is available in the Supplementary File S1. Furthermore, in addition to calculating the number of shared IBDs between populations, we also calculated the average and median lengths of these IBD fragments for every pair of populations. This data is shown on additional tabs in the Supplementary Table S1.

### 2.2. Modern Genomes

**Figure 2:**
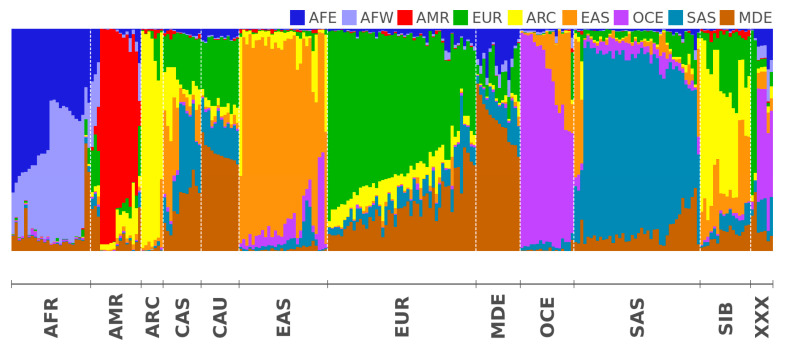
The graphical stacked-bar representation for the Supplementary Table S4. Each bar is a population. The color and the height correspond to the percentage of IBD shared with the corresponding geographical region. The colors denoting geographical regions are given at the top of the graph. The populations are grouped into 12 geographical regions which IDs are given at the bottom of the graph. The populations are ordered within each geographical region by the percentage of shared IBD fragments.

Detailed analysis of Supplementary Table S1 reveals that there are several world regions in which the population appears to have been genetically detached for a long period of time, where people from local populations only share high amounts of IBD fragments with one another. In other words, when these specific local populations are compared to any other populations from any other region, the numbers of shared IBD fragments are many times lower. For example, Arctic populations from Northern Eurasia and North America (Eskimos, Koryaks, Chukchi, etc.) share about 20 times more IBD fragments with each other than with populations from China, Europe, or Central Asia. We empirically identified nine of these world regions as Distinct Human Genetics Regions (DHGRs) or regions whose inhabitants share many times lower amounts of IBD fragments with every other region besides themselves. Within each of these DHGR, we identified three reference populations using criteria of populations that are the most unique and share the least number of IBD fragments with the rest of the World. These populations presumably have been genetically detached for hundreds of years and did not experienced significant admixture with the rest of the world. DHGR regions and their reference populations are described in Table 3. Finally, for every population we studied, we calculated the relatedness for the population under analysis with nine DHGR groups shown in percentages (see Supplementary Table S4 and its graphical representation on Figure 2). These data indicate the genetic admixture/relatedness for each of the studied populations with nine DHGR groups.

In contrast to the described genetically detached populations, Table S4 demonstrates how several populations in the middle of Eurasia represent complex admixtures, defined by sharing relatedness with four DHGR components above 10%. We detected the most heterogeneous patterns of admixture in Uyghurs (China/Kazakhstan), with a genetic relatedness to East Asia (EAS) of 28%, Europe (EUR) 21%, Hindustan Peninsula (SAS) 17.5%, Middle East (MDE) 17%, Arctic (ARC) 13%, Oceania (OCE) 2%, and Africa (AFR) 1.5%. The top admixture in the Asian continent can be observed among the Uzbek population from Central Asia (Uzbekistan Republic), Azerbaijanians, Iranians, and Pathan people from Pakistan. These populations lived for thousands of years in the middle of a complex network of trading, migration, and conquest events where millions of people representing the world’s vast spectrum of genetic diversity passed through. Due to a historically high number of admixed people and large population sizes, the numbers of shared IBD fragments within the same population in the aforementioned groups are the lowest compared to the rest of the world (for Pathans, their shared number of IBDs among themselves is the record low—8.95; this is followed by Azerbaijanians and Uyghurs, each at 10.8; Uzbeks at 11.1; Iranians at 12.2; and Turks at 12.9). Together, the low number of shared IBDs within the same population and the high values for relative relatedness from multiple DHGR components (Supplementary Table S4) indicate the populations with the strongest admixtures where millions of people from remote populations mixed with each other for hundreds of years. In Europe, such admixed populations include the Moldavians, Greeks, Italians, Hungarians, and Tatars, among others. In the Americas, the highest admixture was detected in the Mexicans from Los Angeles (MXL) and Peruvians (PEL) presented by the 1000 Genomes Project.

In contrast, small populations composed of only several thousand people (e.g., from Oceania) have been severely exposed to inbreeding and as well as founder effects. A majority of individuals within these populations are relatives of one another. This intra-populational relatedness is manifested in the highest numbers of shared IBD fragments within the same population. These numbers are often higher than 100 IBDs per random pair. We observed the highest number of shared IBD segments within the same population in Oceania (Igorot, Philippines 247; Koinanbe 244 and Bougainville 240 from Papua New Guinea). We also saw similar numbers in small populations from South America (236 Wichi, Argentina), Arctica (222 Eskimos), and Africa (221 Ju Hoan in Namibia, 212 Khomani in South Africa, and 190 Pygmies in Congo). Due to the effect of inbreeding, the number of shared IBD fragments between these small populations and neighboring populations may be high as well. For example, the Bedouin intra-population number of shared IBDs is very high (176), while the intra-populational IBD sharing number of its much larger neighbor, Iran, is only 12. This is a large difference; however, the number of shared IBDs between Bedouins and Iranians is 20 (nearly twice the number found when evaluating the Iranian population by itself). This effect is due to elevated inbreeding among Bedouins, which makes a fraction of very rare SNP alleles much more frequent among Bedouins (and subsequently, Iranians after their admixture with Bedouins).

### 2.3. Ancient Genomes

There is a strong distinction between the characterization of very rare SNP alleles in ancient genomes versus modern genomes, especially when taking into account that Neanderthal lineages separated from modern humans more than 500,000 years ago. Due to this extended period of separation, Neanderthal groups should have their own pool of very rare SNP alleles, which must differ significantly from the pool of rare alleles in modern people. However, because there are only a handful of Neanderthal/Denisovan genomes currently available for genotyping, it is impossible at this time to deduce any very rare alleles from these ancient people. Therefore, we considered all the alleles of ancient people that matched the very rare SNP alleles found in modern peoples as “very rare SNP alleles” of ancient people. Thus, when evaluating this paper, a reader should keep in mind that many of these alleles might have actually been frequent alleles among Neanderthal/Denisovan genomes. When a small group of Neanderthal/Denisovans admixed with much larger populations of the ancestors of modern peoples thousands years ago (as has been suggested by multiple publications [5,17,18,19,20], all the frequent alleles found in the Neanderthal lineages were diluted and consequently became rare alleles among our ancestors. As a result of this dilution, Table S1 exhibits very high numbers of shared IBD fragments between ancient and modern peoples. The highest numbers of shared IBDs is observed between Oceania populations and Neanderthals/Denisovan, where they sometimes exceed 80 IBD fragments per pair. The detected overabundance of Neanderthal genetic contributions compared to Denisovan genetic contributions in Oceania aligns with evidence found in recent publications [20,21]. Such uncharacteristically high numbers may suggest an accidental match (Identical-by-State instead of IBD) because of the large pool of “very rare SNP alleles” in ancient genomes in our calculations (e.g., Neanderthal Cha_XXX has 182,218 very rare SNP alleles in our dataset). In order to investigate the possibility of a random match, we used computer modeling to analyze the data by generation of “random Neanderthal genomes” using our program randomENTRY_vrGVdb.pl (available in Supplementary File S2). Each random Neanderthal genome was created by using a random number generator to pick very rare alleles from the entire very rare allele pool of all modern people. From our calculations, we required that each “random Neanderthal genome” had the same number of very rare SNP alleles as the real Neanderthal genome. Supplementary Table S1 contains an example of two “random Neanderthals” with identifiers RND_XXX and Rnd_XXX. On average, we saw only 15 shared IBD fragments between our “random Neanderthals” and our group of 3121 modern people. In contrast, real Cha_XXX Neanderthal shared more than 42,000 IBD fragments with the same set of modern people. Thus, the probability that shared IBD segments observed between prehistoric genomes and modern peoples occurred by chance is only 0.03%, which is well below the cutoff of significance used to define our results.

**Figure 3:**
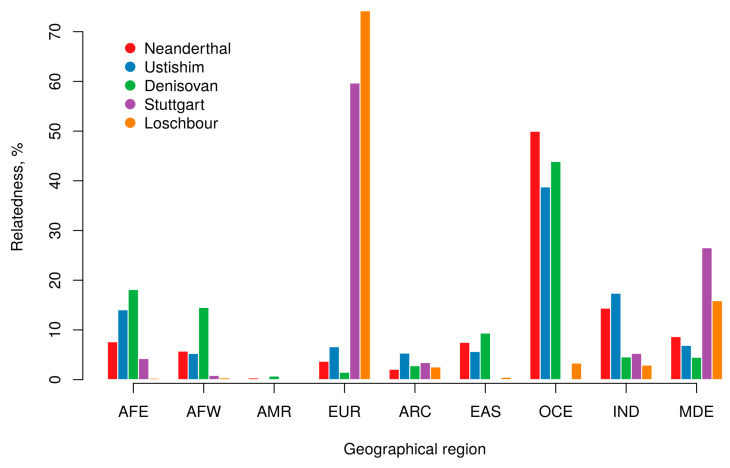
Relative abundance (in percentages) of IBD fragments from prehistoric people in modern people from major geographic regions.

The genomes of all three studied Neanderthals (Altai Neandertal (Denisova5, Alt_XXX), Chagirskaya (Cha_XXX), and Vindija (Vin_XXX)) have strikingly similar patterns of relatedness to modern populations, despite these prehistoric people having lived ~30,000 years apart from each other in the area from southern Europe to Western Eurasia and Altai (see bottom of Supplementary Table S4). Supplementary Table S4 demonstrates that Neanderthal genomes have the highest percentage of relatedness (around 50%) with people from Oceania, followed by South Asia (~15%). Meanwhile, American and Arctic populations have the lowest Neanderthal relatedness (~0.5% and ~2% respectively). Similar to the Neanderthal genome, the Denisovan genome (Den_XXX) also had the highest level of admixture with the Oceania populations. However, Den_XXX contains much higher levels of relatedness to African genomes (see Figure 3). In fact, around 30% of the Denisovan genome relatedness to modern humans comes from Eastern and Western Africa. Drastically lower percentages of Denisovan relatedness to Hindustan and Middle East populations is another notable difference between Denisovans and Neanderthals. There are distinct patterns in the estimated relative relatedness for one of the oldest humans known to science—the Ustishim man from Siberia—with modern humans (see Figure 3). These patterns fall in line with recent publications [22,23]. Thus, all three Neanderthals were clustered together and categorized as representatives of the same group, while Denisovan archaic lineage and the Ustishim prehistoric modern human belong to different groups.

Analysis of ancient humans from Europe who lived 7000–9000 years ago demonstrated a higher impact of the Luxemburg’s Loschbour genome (Los_XXX) on modern Europeans than the Stuttgart (Lbk_XXX) genome (~74% and ~60% relatedness respectively, see bottom of the Supplementary Table S4). In contrast, the relatedness to modern Middle Eastern (~27% vs. ~16%) and East African (~4% vs. ~0.3%) genomes was found to be higher in the Stuttgart ancient human than in the Loschbour human. Such differences in genomic impact between Loschbour and Stuttgart ancient humans on modern populations are consistent with their affiliation to two distinct human cultures: Western hunter–gatherers and the first European farmers, respectively [24]. The European farmers originated from the Near East, (e.g., Anatolia, Turkey) and they started to spread across Europe only during the Neolithic period [25]. The relative presence of Archaic/Ancient genomes in modern humans is shown in Figure 3.

### 2.4. Time Periods for IBD Sharing between Populations

**Figure 4:**
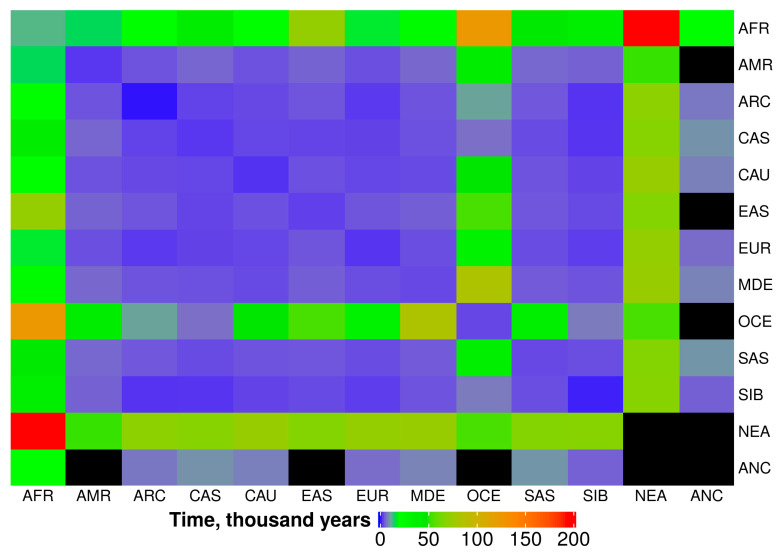
Heat map for estimated age of IBD fragments which are shared between populations from major geographical regions. Black color represents N/A data.

The genetic relatedness between a pair of people manifests itself through the presence of shared IBD fragments within their genomes. The size of a shared IBD is, on average, inversely proportional to the time that has passed from the last common ancestor who transmitted this IBD. Because the passing of IBDs from generation to generation is a stochastic process, the distribution of IBD fragments by size has a very wide, unimodal shape. Therefore, calculating the time of appearance of shared IBDs requires rigorous statistics and the use of a median or average length of shared IBD fragments between populations [1]. In this study, we calculated medians and averages for the IBD fragment lengths for every pair of compared populations (see Supplementary Table S1, IBD-Medians and IBD-Average tabs). The smallest IBD sizes compared to other groups were observed for Neanderthal, African, and Oceanian populations. We also integrated this large dataset of IBD medians for each pair of populations (Table S1) to the medians for 12 larger geographical groups (Table 4). We detected the smallest median IBD sizes between Africans and Oceania populations (17 kB), Neanderthals and Africans (22 kB), and East Asia and Africans (29 kB). During this IBD length analysis, we detected a deviation from the unimodal IBD-length distribution when comparing the East Asian vs. Oceania populations, which was represented by two peaks for IBD lengths. One peak was observed for each studied population from Papua New Guinea (Koinabe, Kasipe, and Papuans) and native Australians with the 39 Kb IBD median for these four populations vs. EAS (Table 4 and Table 5 and Figure 4). On the other hand, the rest of the Oceania populations (from Philippines, Indonesia, etc.) presumably had more recent instances of admixture with East Asian populations, as their total median IBD length with East Asia is 454 Kb, which is eleven times larger than for Papua New Guinea representatives. For this reason, only the Papua and Australian populations represent the “OCE” data in Table 4 and Table 5 and Figure 4).

We go into further detail of the relationship between median IBD lengths and the time they have been shared between populations in the Methods and Discussion sections. We made these calculations based on conventional formulas, which were further calibrated by taking into account several known historical events applicable to our dataset. The time period for IBD sharing between populations (history of admixtures) is shown in Table 5, while a heat-map of these data is depicted in Figure 4.

## 3. Discussion

### 3.1. Problems with Short IBDs and Estimation Their Time of Origin

Comparisons of neighboring populations result in the detection of large, (around 1000 Kb, or 1 Mb) shared IBD fragments. In contrast, comparisons between populations from different continents or between archaic and modern peoples produce much shorter IBDs. These short IBDs pose significant problems for genetic interpretation. For example, when we compared archaic peoples with African populations, we observed the median size for shared IBDs to be 22 Kb long. This short size contrasts with the even shorter IBD size estimated by Povysil and Hochreiter, who conducted an earlier analysis on the same groups [26]. Their IBD distributions had a median of around 8 Kb. We acknowledge that our approach is likely to miss a considerable portion of the very short (<10 Kb) IBD segments. This is because there is only about one very rare SNP allele per 10 Kb in the human genome. We decided not to loosen our stringent criteria for characterization of IBDs (which is at least 5 very rare SNPs) because false positive results with these conditions are negligible (see our computer modeling with Rnd_XXX in the Section 2). Therefore, our approach yields a cleaner background but likely creates a bias by missing many of the shortest IBD fragments. Altering this constraint (for example, changing our lower limit to a cluster of three very rare SNP alleles instead of five) would very likely help in detecting the shortest IBDs, but would also greatly increase the false positive IBD detection. This sort of “loose criteria” was used in the study conducted by Povysil and Hochreiter [26]. Their paper considers SNPs with the minor allele frequency as high as 5% (instead of the much more conservative 0.3% that we used in our project). Consequently, the length distribution of their IBDs is dominated by a large number of very short (<5 Kb) fragments. More likely than not, these short fragments include false positive results within the reported range.

In summary, IBD fragment characterization has a natural threshold at around 20 Kb in length. At this threshold, we encounter a significant dilemma: Either losing a significant proportion of short IBDs, or including a large assemblage of false positive hits. The foundation for this dilemma is arguably trivial. The shortest DNA fragments do not host enough polymorphic sites for their undisputable identification. As just noted, we made our criteria highly stringent to greatly reduce the amount of false positive data. Inescapably, it is likely that we missed a substantial number of very short IBD fragments. However, given the choice between these two, a high threshold for rare alleles is more likely to yield misleading results than is the exclusion of the very shortest IBDs.

Another problem that requires consideration is the chronological placement of the Last Common Ancestor (LCA) based on IBD lengths. The theoretical formula for the conversion of average length of shared IBD fragments into the rough date for their LCA is: ***L =* 1/(2 *× g × r*)**, where ***L*** is the average length of IBD fragment, ***r*** is the meiotic recombination rate, and ***g*** is the number of generations since LCA [26,27]. The biggest problem here is that the recombination rate along genomes is tremendously uneven, differing by as much as three orders of magnitude [28]. Moreover, meiotic recombinations frequently occur at the same recombination “hot-spots”. Multiple occurrences of recombination events in the same site do not have any effect on IBD length. This means there is likely to be significant deviation in calculations of real time for the LCA from the aforementioned formula. The correct placement in time of the LCA is thus a very difficult problem—one that is outside the scope of this paper. Accordingly, our calculations of LCA times in this study represent a “best guess” based on calibrations of theoretical computations, using well-established historical events as described in Methods section. Our computed times for the LCA datasets are presented in Table 5 and Figure 4. All of these LCA calculations are linked together pretty rigidly, when viewed in relation to one another. In other words, one demographic event characterized with shorter IBDs must precede another one, which was characterized with longer IBDs. Additionally, it is important to note that the entire scale of events might be shifted forward or backward in time due to the described uncertainty. To support our considerations in this paragraph, we would like to quote Povysil and Hochreiter, who very appropriately wrote: “We are not confident in absolute age estimations of the IBD segments based on their length” [26].

### 3.2. Modern Genomes

Our results concerning the relatedness of modern people generally align with previous evolutionary studies based on the same datasets of the 1000 Genomes, Simons SGDP, and Estonian EGDP projects, which were processed and obtained through different methods in the original publications [4,7,8]. A major criterion, that reliably if unsurprisingly indicates significant genetic relatedness between populations, is whether the populations historically occupied neighboring territories. For example, Iranians (Persians) emphasize Indo-Europeans as the base population for their genetic origins [29]. Furthermore, for thousands of years beyond Biblical time, Iranians have been in close contact with inhabitants from the Middle East. Based on the aforementioned criteria, it is unsurprising that modern Iranian people have the highest (48%) relatedness to MDE people and significantly lower relatedness to SAS populations (20%) and EUR (12%) (Supplementary Table S4). Another example is Caucasian populations. In this region, there are hundreds of ethnicities with their own languages and religions. These populations clearly distinguish themselves from each other. Current data shows that all Caucasian populations have very similar patterns of relatedness: The strongest relatedness is to MDE (~45%, on average), then EUR (~27%), and SAS (~14%) with only slight variations from one Caucasian native to another (Supplementary Table S4). In addition, there are no clear differences between North and South Caucasus groups which have been described [30]. To a certain extent, they can be seen in the proportions of relatedness between Caucasus populations and EAS group. For example, Kabardians, Circassians, and Ossetians have noticeable levels of EAS related IBDs (up to 5%), whereas other Caucasian groups have much lower (1–2%) relatedness to EAS. This suggests ancient admixture/migration events that occurred within specific populations in Caucasus. Their source could be associated with the steppe populations [30].

Regarding Europeans, the strongest genetic distinction in their population occurs along the North-South axis. All European Mediterranean countries (Spain, Italy, Greece, Albania, Croatia) have around the same level of relatedness to the Middle East and North Europe (around a 1:1 ratio at 40%, see Supplementary Table S4). In contrast, the Middle East component of relatedness in Scandinavians and other people from Northern Europe is lower than 10%. Taking into account both the results of our comparisons of relatedness between the Loschbour and Stuttgart genomes and modern genomes and the data of other studies, such general south-east cline seems to be correlated with the intensity of migrations of Neolithic humans (farmers) and their admixture with local hunter–gatherer populations. Their lower intensity in the Northern parts of Europe resulted in the preservation of higher level of initial hunter–gatherer ancestry in modern-day populations [25,31].

In addition, we see a distinction between European populations along the west–east axis, which is exhibited through their relatedness to the Arctic group. In the north-east regions, the relatedness of Europeans to Arctic groups reaches 15%, while in Western Europe, it drops to around 5%. As it has been recently proposed, this pattern is associated with the gene flow from Western Siberian populations [22]. All of these populations traced up to 60% of their IBD fragments to Arctic group.

Among 47 studied European populations, only one (Roma, Bosnia-Herzegovina) stands out from the others by its unique pattern of genetic relatedness. Specifically, the Roma population has the highest genetic relatedness (44%) to the SAS natives, which is, on average, eight times higher than in other Europeans. The Roma people came to Europe from India, and astonishingly, they have almost fully preserved their genetic identity despite producing dozens of generations since their separation from their original founder population. The Hungarian ethnic group represents the opposite situation. Hungarians are the only people in Central Europe who speak a language belonging to the Uralic language group instead of Indo-European, like all other peoples of this region. It is well-known that the ancestors of the Hungarians initially lived in the steppe regions of the Ural region, and their closest relatives were the ancestors of the modern Khanty and Mansi [32,33]. In the first millennium AD, these ancestors of the Hungarians began to move westward and reached what is now known as modern-day Hungary in Europe. At the time, this territory was inhabited by Slavic and Germanic tribes, and the Hungarians fought with these peoples to eventually claim the area. During their conquest of these lands, the Hungarians mixed with representatives of the local Slavic and Germanic tribes, changing the genetic composition of their population. Thus, according to genetic data, Hungarians became a typical Central European ethnic group, yet they were able to retain their language through social and geographical dominance. According to our data (Supplementary Table S4), Hungarians’ similarity with the Germans (Ger_Eur) and Croats (Cro-Eur) is extremely high, but there is a significant difference from their language relatives—Khanty Kha_SIB and Mansi Man_SIB.

As for American populations, the smaller ethnic groups, recently characterized in the Simons SGDP and Estonian EGDP projects in South America, did not show significant levels of relatedness (<2%) to either Europeans or African populations. In contrast, American populations, first characterized within the 1000 Genomes Project, all have predominant relatedness to Europe, Middle East, and Africa. Interestingly, the Americans from the 1000 Genomes Project demonstrated almost the same level of relatedness to Europeans and to Middle Easterners (Supplementary Table S4). It happens because most admixture of these American populations with Europeans resulted from their contact with people from Spain, Portugal, and Italy, which has about 50/50 relatedness to European and MDE groups, as it was described in the previous paragraph. At the same time, they had the least relatedness to the South American populations delineated in the Simons SGDP project. In fact, Puerto-Ricans (PUR) have the lowest relatedness (1.5%) to the AMR group, followed by Colombians (CLM) with only 3.7% of AMR relatedness, and finally Mexicans from Los Angeles (MXL) with 11% of AMR relatedness (Supplementary Table S4). In contrast, Mayan, Pima, and Mixe people from Mexico, characterized within the Simons SGDP project, have much stronger relatedness to the reference Native Americans (AMR) (80%, 67%, and 65% respectively). This points to the commonality in basal ancestry of present-day Native Americans. Additionally, comparison of the patterns of relatedness demonstrates substantial diversity between North and South Americans. In particular, Northern Americans preserve more ARC related IBD segments. This observation may reflect the diversification of ancestral Native Americans into Northern and Southern branches that occurred around 13,000 years ago [34]. The genomic impact of other populations (including populations from Oceania) on Amerindians is significantly lower. From this, we can hypothesize that either the Australian related gene flow to ancestry of South Americans was more or less insubstantial [35] or that the gene flow was very old.

The populations of the Arctic (ARC) are strikingly distinct from their much larger and more populated neighbors. We were surprised to see only 1% of relatedness between both Koryaks and Chukchi populations to the EAS group. In addition, many ARC populations have less than 1% of relatedness to Europeans. At the same time, the strong genetic contribution of the Arctic group (up to 50%) has been detected in several ethnic groups in Siberia as well as in small populations from Northern China (Supplementary Table S4). This highlights the specificity of the genomic history of ARC populations and demonstrates a strong correlation with data from recent studies on the demography of Northeastern Siberia. In these studies, the demography of northeastern Siberia was proposed to be populated in three expansion events, one of which gave rise to the ancestors (Ancient Paleo-Siberians) of both the ARC group and Native Americans [23]. The data on the latter is in strong accordance with our data on substantial relatedness between ARC and Native Americans. In the mid-Holocene period, Ancient Paleo-Siberians were largely replaced by/admixed with the third wave of East Asian related migrants—Neo-Siberians, from whom many modern Siberians are descended [23]. The variation in patterns of relatedness between the Siberian populations and ARC and EAS groups that we observed in our study reflects the local differences in admixture, resulting in mosaic genetic make-up of contemporary people of Siberia. In addition, we detected the ARC related identity in European populations, particularly in Northern Europeans. However, it should be noted that the Europeans seem to have received the ARC ancestry component indirectly through admixture with groups related to modern Western Siberians [22]. Arctic populations are the most prominent group that admixed with native Americans in pre-Columbian time. These people have long been living in coastal regions by the ocean and are experienced seafarers. Their use of sea routes eventually led to the widespread expansion of their genetics all throughout the Arctic [31]. The peak of the Arctic populations’ admixture with Native Americans is around 5200 years ago according to our calculations. Additionally, we detected a previous admixture of the Arctic group with Oceania nations around 12,000 years ago.

We acknowledge that the most common approach for studying African populations is accomplished through comparing populations from the North and South African regions [36,37]. We saw a very strong admixture of Northern African populations with primarily Middle Eastern and secondarily European populations. Meanwhile, this admixture is virtually absent south of the Sahara Desert. This regional difference in admixture is well-known, therefore, we decided to make our main focus the largely unexplored West-to-East genetic variability of populations within the African Continent [38]. Thus, we separated and compared the data from both West and East Africa regions, and this became one of our primary focuses of the continent (Supplementary Table S4). Our data demonstrates that the largest impact on African relatedness to other populations is seen in the Middle East where the relatedness from East Africa reaches 20%. Beyond the Middle East, the noteworthy relatedness (about 5%) is observed to be spread throughout the neighboring Caucasus. Southern Europe is another region that contains populations with strong genetic relatedness to African populations: Relatedness reaches up to 10% in Spain and 7% in Italy.

Oceania populations represent an interesting case because they separated from the mainland around 40,000 years ago according to our estimations (Table 5). The exceptions to this case include Arctic, Siberian, and Central Asia populations, which admixed with Oceania groups about 8000 years ago. We were able to register Native American contacts with Oceania populations that occurred roughly 40,000 years ago, which predates the apparent human migration across the Aleutian land bridge (~14,500 years ago [39]). The most ancient relatedness observed in our project between modern peoples was found for African-Oceania populations, which occurred around 125,000 years ago.

We tried to reveal genetic traces of Medieval global migrations across Eurasia associated with the expansion of the Genghis Khan empire, which was created in 1206 and eventually spread from China to just outside of Central Europe. Russia was one of the regions occupied by Mongols for about three centuries. Tatars, the presumable descendants of Mongols, and many of their neighboring populations demonstrate surprisingly small relatedness (3–5%) to East Asia. For the modern Mongolian population, the EAS people are the dominant relatedness component (60%, see Supplementary Table S4). Among Russians, the EAS component is very small (1.2%), and this finding is comparable to many populations from Central and Western Europe. Conversely, in Central Asia the genetic relatedness to East Asia is one of the major components. This relatedness reaches 35% in Kyrgyzstan and Kazakhstan as well as among Altaians and Kalmyks. In the Middle East, the genetic relatedness to East Asia is negligible (mostly <1%) with the exception of Turkish people, where it reaches 4.3%.

### 3.3. Ancient Genomes

As we explained in the Section 2 there is a specific limitation affecting our definition of very rare alleles among archaic/ancient genomes. Specifically, all alleles matching very rare alleles in modern people, are denoted automatically as having been very rare alleles in the archaic/ancient people. This approach allowed the detection of a vast number of IBD fragments that have been retained by modern humans from their prehistoric ancestors. It should be also noted here that although several thousands of ancient genomes have been characterized only few of them were sequenced with high coverage (×20) which is appropriate for studying whole genome variation [15]. Many papers on the Neanderthal/Denisovan genomes discuss how it is most closely related to the genomes of populations in Oceania [17,20,40,41,42]. Our data (Supplementary Table S4) also demonstrate that the major relatedness component of Denisovan genome to modern people is observed in Oceania populations (44% for OCE). In addition, all three studied Neanderthal genomes (Alt_XXX, Cha_XXX, and Vin_XXX) have even higher relatedness (~50%) to Oceania as well. Examination of individual IBD fragments shared between Neanderthal or Denisovan with modern Oceania genomes demonstrated that at least two thirds of these shared IBDs are common to Neanderthals and Denisovan (results not shown yet presented in Supplementary Table S3). Therefore, we were unable to distinguish whether “prehistoric” chromosomal fragments in Oceania genomes were descended from Denisovan or Neanderthal lineages. Several papers demonstrated an additional strong relatedness of archaic man to African populations [5,43,44]. We also clearly see this effect in Supplementary Table S4, where Denisovan relatedness to East Africa is 18%, while to West Africa it is 15%. Detailed analysis of African/Denisovan relatedness from Table S1, shows that it predominantly comes from populations in Central Africa (Pygmies and Mbuti, Congo; Biaka, Central African Republic) and Bushmen people from South Africa (Ju-Hoan Namibia, and Khomani San, South Africa), which is similar to the data from Wall and co-authors [5]. In addition, we also confirm through an observation of Wall and others that a pull of shared IBD fragments between Denisovan and Oceania practically does not intersect (<10% intersection) with the pull of shared IBDs between Denisovan and the described African populations (Supplementary Table S3). Our IBD analysis detected noticeable relatedness of Neanderthal/Denisovan genomes to SAS populations (up to 15%), MDE (up to 9%), EAS (up to 9%), EUR (up to 4%), and ARC (up to 3%), while only 0.5% to AMR (see Figure 3). We observed very small levels of admixture between Native American populations and Neanderthals/Denisovan (Figure 3). We only detected 12 shared IBD fragments (out of 27 possible pairs) between 3 Neanderthals and 9 Americans from three reference populations in Table 3 (Karitiana, Piapoco, and Wichi). In addition, we observed 5 shared IBDs from the same groups of Native Americans and Denisovan. These shared IBDs are from five genomic sites (Chr1, starting position: 167,592,916; Chr2, position: 155,342,689; Chr8, position: 3,469,769; Chr10, position: 107,842,347; and Chr10, position: 126,909,100). We also detected all five of these IBDs in East Asia and Siberia, however, none of them were detected in Oceania and Africa. Our data may differ slightly from data described in other publications on Neanderthal/Denisovan DNA in Native Americans [19]. Consequently, this issue may be motivation for future detailed investigation.

The most recent admixture of Neanderthals/Denisovans was observed in Oceanian populations and occurred ~55,000 years ago. Following this admixture was the admixing of Europeans and a large spectrum of Asian populations about 65 to 75 thousand years ago. While admixture of archaic people with Africans occurred much earlier (around 190,000 YA). Additionally, African–Neanderthal shared IBD fragments are among the shortest (~22 Kb median). The detection of such small IBD fragments requires special analysis, and this is why the Neanderthal–African relatedness has not been detected in several publications. In fact, given the strict criteria we used (discussed above), this is near the lower size limit of what we could reliably detect in our analysis.

As for genomes of ancient individuals who lived around 7000–9000 years ago in the vicinity of Luxemburg (Los_XXX) and Stuttgart (Lbk_XXX), we see the progenies of the former spread primarily within Northern Europe, while the latter spread in the Mediterranean region and then the Middle East as well (see Figure 3).

## 4. Conclusions

Analyzing clusters of very rare SNP alleles to accurately detect numerous shared IBD fragments within diverse, whole-genome sequencing datasets is an instrumental method in piecing together a cohesive global picture of human genetic relatedness. This approach allows for the detection of very short IBD fragments (up to 10,000 nucleotides), which may be over 200,000 years old and difficult to harness due to their shorter size. Resolution of such small IBD fragments enables the detection of genetic relatedness between various remote populations from different continents. We were able to uncover human populations with the highest and lowest admixtures, as well as trace the inheritance of IBD fragments from seven archaic and ancient peoples to modern genomes all over the globe. Relative relatedness of every studied population to nine distinct human genetics regions has been calculated. Further unravelling the peculiarities in the origin and evolution of human populations boils down to simplicity, explicitness, and the opportunity for continued in-depth analysis of individual IBD fragments.

## 5. Materials and Methods

All of the main principles, protocols, and programs used in the creation of the rare SNP alleles database have been described in detail in Fedorova et al. [1]. Here we present a major upgrade to our project, which involved combining four whole-genome sequence datasets to increase the number of populations from 14 to 232. The following whole genome sequence datasets were used for this upgrade: (1) The 1000 Genomes Project (phase III), which included 2504 individuals from 26 populations and was downloaded in VCF format (ftp://ftp.1000genomes.ebi.ac.uk/vol1/ftp/release/20130502); (2) the Simons Genome Diversity Project included 279 genomes from 142 populations [8]; (3) the Estonian Biocentre Human Genome Diversity Panel with 402 new genomes from 125 populations [4]; (4) the whole-genome sequencing data of 27 modern-day humans from Siberia and Western Russia by Wong EH and co-authors [22] (named here as Siberian Project) were downloaded from Sequence Read Archive with fastq-dump tool of SRA Toolkit 2.9.2 (https://www.ncbi.nlm.nih.gov/sra accession number SRP053199). To obtain SNPs, we processed fastq files with a custom pipeline. It consisted of the following steps: Estimating the quality of sequencing data with fastqc v0.11.8 [45], mapping the raw reads to the Human reference genome of GRCh37 assembly with bwa mem 0.7.17-r1188 [46], sorting and indexing the aligned reads with samtools 0.1.18 [47], estimating the coverage with mosdepth 0.2.5 [48], calling for variants with freebayes v1.2.0-2-g29c4002 [49], filtering the variants with vcffilter from vcflib, normalizing the variants with vt [50], removing indels with vcftools 0.1.15 [51], and annotating the variants by dbSNP151 with SnpSift [52]. The SNPs of 18 individuals from this dataset were involved in further analysis. Ninety-seven people with close relatedness from the 1000 Genomes Project have been removed from our analysis.

In this research we also analyzed SNPs from seven ancient genomes. They represent two archaic human groups (Neanderthals and Denisovans) and anatomically modern humans leaved 45,000 (Ustishim) and 7000–8000 (Stuttgart and Loschbour) years ago (Table 6). The SNPs were downloaded in the format of VCF files from the repository of Max Planck Institute for Evolutionary Anthropology http://ftp.eva.mpg.de/neandertal. We included these samples in our research because of high coverage of sequencing available.

Shared IBD segments between a pair of individuals were identified as clusters of at least five very rare neighboring SNP alleles that are common to both individuals. As previously demonstrated, the threshold of five neighboring very rare SNP alleles reduces the chances (*p*-value < 10^−13^) of any possible sequencing errors to nearly zero, which may mimic real IBD fragments and create false positive IBDs [1].

Prior to starting computational processing of the datasets, we tested different frequency thresholds for very rare alleles. Based on these trials, we increased our initial threshold for very rare SNP allele frequency from 0.2% to 0.3% across all populations. This new threshold allows for the analysis of a much greater number of IBD fragments while still maintaining the ability to prevent most of the noticeable noise from the accidental arrival of Identical-by-State chromosomal fragments. With this current upgrade, the core database of very rare SNP alleles was created based on the 1000 Genomes Phase III dataset in accordance to Fedorova et al. 2016 protocols (using the updated version vrGVdatabaseATLAS2_A2.pl of our Perl program) [1]. This initial database contains a core set of 39,634,907 SNPs representing very rare alleles (vrGV_1KG_A2 database). All the remaining SNPs from the 1000 Genomes Project that did not represent these very rare alleles, were placed into a complementary database of so-called “NON-very-rare” SNP alleles. This NONvrGV_A2 database contains 20,625,576 SNPs. Three other genome sequence datasets, which we further processed during this project, contain a significantly lower number of genomes than the 1000 Genome Project. A reliable characterization of very rare alleles is a major challenge that these small datasets face. To resolve this problem, we compared every SNP from these three databases with SNPs from NONvrGV_A2. When a SNP allele under analysis matched a SNP from NONvrGV_A2, it was automatically rejected. This criterion was introduced to reduce potential batch effect associated with using different databases and sequencing technologies. However, if the SNP passed this initial filter, we calculated its allele frequency in the current genome sequence dataset. When a given minor allele occurred four or less times in the Simons SGDP or Estonian EGDP projects, or one or two times in the Siberian Project, it passed the second computational filter and was added into our database of very rare SNP alleles. These computations of very rare SNP alleles were performed by three Perl programs specific to each database (vrGVdatabaseSIMON2_A2.pl; vrGVdatabaseESTONIA2_A2.pl; vrGVdatabaseVALUEV_A2.pl). After updating the very rare SNP alleles database using all four genomic projects, we processed the seven archaic and ancient genomes. We compared all the alleles of these prehistoric peoples to our known very rare SNP alleles, and when there was a match, the prehistoric SNP allele was added to the database. Each of these archaic/ancestral genomes added from 22,977 (Ustishim prehistoric modern human) to 182,218 (Chagirskaya Neanderthal) very rare SNP alleles to our final database. Note that these alleles may not be very rare for archaic genomes (as we discussed in the Results section of this paper). The final version of the very rare SNPs database was named vrGVdb_1K_S_E_V_A7_all_v3, and it contains 65,957,935 very rare SNP alleles. vrGVdb_1K_S_E_V_A7_all_v3 is publicly available as Supplementary File SF1. Finally, this database also contains two computer-simulated archaic genomes (identifiers: Rnd_XXX and RND_XXX), whose very rare alleles have been created by a random number generator that picked alleles from vrGVdb_1K_S_E_V_A7_all_v3. The Perl program randomENTRY_vrGVdb.pl was used for creating these simulated genomes. The Rnd_XXX simulated genome had 182,000 very rare SNPs (the same as Chagirskaya) while the RND_XXX had 87,000 (the same as Denisovan Pinky). Auxiliary files that include the names of all individuals, populations, and geographical regions, which were used by our pipeline of Perl programs are included in the Supplementary Materials.

We created special identifiers for the individuals, populations, and geographic regions under analysis. Population identifiers were constructed using two groupings of three characters (triplets) that were concatenated together by an underscore symbol. The first triplet is related to the population name, while the second refers to the geographic region of the respective population. For example, Rus_EUR is the identifier for the Russian population from Europe, while Rus_CAS represents the Rushan-Vanch population from Tajikistan (Central Asia). We considered 12 geographical regions: AFE—East Africa; AFW—West Africa; AMR—America; CAS—Central Asia; CAU—Caucasus; EUR—Europe; ARC—Arctic region; EAS—East Asia; SAS—South Asia (within the Hindustan Peninsula); OCE—Oceania and Australia; MDE—Middle East, and SIB (Siberia). In our classification SAS is represented by populations from India, Pakistan, Bangladesh, and Myanmar, while Indonesia and Philippines were assigned to the OCE region followed by Simons SGDP project publication [8]. The geographical identifier for prehistorical ancient peoples is denoted by XXX because these peoples lived thousands of years ago and might have participated in unknown long migrations. Finally, our identifiers for individuals are constructed by linking their population identifier (via underscore symbol) to the identifier for the individual from the corresponding whole-genome sequence datasets. For example, Rus_EUR_LP6005441-DNA_G10 or BEB_SAS_HG03802.

All of the results presented in this paper were obtained through computer processing of vrGVdb_1K_S_E_V_A7_all_v3. In the initial stage of this processing, we obtained individual-specific databases of very rare SNP alleles using the updated program vrGVindividualDBS_A2.pl as described in Fedorova et al. 2016. During the second stage, we obtained “dat3” and “dat4” files for each individual using an updated version of the program, RVC_v3_A2.pl. This version of the program was automatically launched for each individual by the TotalStat_vrGVs_v4_A2.pl script. In the third stage, we obtained Supplementary Table S1 using the updated version of heat_map5_A2_v3.pl. This program computed every pair of populations and produced the following three tables: (1) Table S1 “IBD Numbers”, which presents the average number of shared IBD fragments per pair of individuals between these two populations; (2) Table S1 “IBD Mean Length”, which delineates the average length of shared IBD fragments between two populations; and (3) Table S1 “IBD Median Length”, which describes the median length of shared IBD fragments between two populations. Median length has been calculated only when the total number of observed IBD fragments between populations was five or more. Otherwise N/A value is present in the IBD Median Length table. For IBD Mean Length calculations no restrictions on the number of IBD fragments was implemented. Using genome sequencing data from different datasets caused the numbers of very rare SNP alleles to vary significantly between projects and from population to population. To account for these variations, we calculated a “Normalization Coefficient’ for each population that compares the relative abundance of very rare SNP alleles in a population to the average abundance of very rare SNP alleles across all populations. These Normalization Coefficients are shown in the last column and row of Table S1 They could be as low as 0.20 and as high as 7.8. The number of shared IBD fragments between individuals is proportional to the characterized set of very rare SNP alleles, which often vary from one whole-genome sequencing database/protocol to another. We used “Normalization Coefficients” to normalize the number of shared IBDs between individuals/populations in order to free them from sequencing factors within different datasets. The data with the normalization of shared IBD data per pair of individuals is presented in Table S1 “IBD Normalized Numbers” and calculated by the formula (1).

(1) $$J_{AB}=I_{AB}/\sqrt{N_{A}\times N_{B}}$$

where:

***I_AB_*** is the number of IBD fragments per pair of individuals from populations ***A*** and ***B***;
***N_A_*** is the normalization coefficient for population A from the HeatMapTable (last row);
***N_B_*** is the normalization coefficient for population B from the HeatMapTable (last row);
***J_AB_*** is the normalized ***I_AB_*** value.

In this formula we used the square root of $\sqrt{N_{A}\times N_{B}}$ because we realized—through testing our normalization on the same populations from the four sequencing datasets—that the populations differed in the number of very rare SNP alleles per individual. Thus, we needed to equalize their shared IBD numbers in order to proceed with our calculations. Using the square root of $\sqrt{N_{A}\times N_{B}}$ in the formula (1) solved this issue by maximally reducing the dependency of the number of shared IBDs from the total number of very rare SNP alleles in the sequencing datasets. Meanwhile, usage of the coefficient (*N_A_* × *N_B_*) in the Formula (1), without taking the square root first, caused a systematic over-normalization with the opposite effect.

The first two rows and three columns in Table S1 describe the analyzed populations. Furthermore, they represent population identifiers (for example: *Ban_AFR* or *YiX_EAS*) and populations names/regions. The first column represents the number of analyzed genomes in the population. These numbers may be followed by letters ‘s’, ‘e’, ‘v’, or ‘se’, etc., which stand for the databases their respective individuals came from (Simons SGDP—(s), Estonian EGDP—(e), Siberian Project—(v), or SGDP + EGDP (se)). For example, for the identifier is 10se, or 1v. If there is no letter, the corresponding population came entirely from the 1000 Genomes Project.

The fourth stage of our computations is unique to this research and was absent in Fedorova et al. 2016. In this stage, we created Supplementary Table S4 using the program *rankingATLAS2_v9.pl*, and the data from the Supplementary Table S1 (“IBD Normalized Numbers”). Supplementary Table S4 presents the percentages of relative relatedness of each population to the nine Distinct Human Genetic Regions (DHGRs) (AFE, AFW, AMR, EUR, ARC, EAS, OCE, SAS, and MDE, see Results section). For each population (e.g., Georgia) the program counts the numbers of shared IBD fragments per pair of individuals for this population with the three representatives of DHGR region and then makes a sum of these three numbers. For example, the for the AFE region, the summing number of shared IBDs will be the following: 0.48 IBDs (per pair for Georgia vs. LWK) + 0.92 (Georgia vs. Din_AFR) + 3.12 (Georgia vs. Mas_AFR) = 4.52 (for the AFE group). And so on for each DHGR group. In order to minimize the Founder effect in our calculations, we created an upper threshold of 100 shared IBD segments for any populational pair. For example, in a calculation of Congo (Con_AFR) vs. LWK, the original value was 151.9, however, with the threshold in place, the program changed the value to 100). Finally, we calculated the relative percentages for all 9 components (AFE, AFW, AMR, EUR, ARC, EAS, OCE, SAS, and MDE) in a way that ensured their sum was always 100%. Ranking data for each population (as presented in Table 2) were also obtained by *rankingATLAS2_v9.pl.*

All of the described Perl programs are available on our website (http://bpg.utoledo.edu/~afedorov/lab/ATLAS2.html) in a package that includes an Instruction Manual and Protocols and also as Supplementary File S2.

To calculate the time period for the last common ancestor, we examined shared IBD fragments based on their lengths using the theoretical formula ***L =* 1/(2 *× g × r*)**, where ***L*** is the median length of IBD fragments, ***r*** is the meiotic recombination rate, and ***g*** is the number of generations [26,27]. In contrast, for the conversion of the average length of shared IBD fragments between ancestral and modern people into the age of their separation we used another formula, ***L =* 1/(*g × r*)**, which lacks a factor of two in the denominator [1]. The factor “2” in the denominator comes from the fact that modern people often share independent IBD fragments, rather than a pair of identical IBDs. We are only able to detect the intersection between these IBDs. On average, this intersection is twice as short as full-length IBDs. To convert the number of generations into historical time, we assume that one generation occurs every 25 years, on average. Both aforementioned formulas would not be accurate because meiotic recombination frequently occurs at the same hot-spot sites with short nucleotide consensuses [57]. In order to take this phenomenon into account, we added a calibration constant (***C***) into the two formulas, resulting in the following variations: ***L = C/*(2 *× g × r*)** and ***L = C/*(*g × r*)** respectively. This calibration constant we established experimentally taking into account the known time period between ancestral men in our study (Los_XXX and Lbk_XXX who lived about 8000 years ago REFS). When considering that, on average, r = 0.0118 Mb^−1^, the median IBD length between these two ancestral people and Europeans is 585 KB, and the fact that Los_XXX and Lbk_XXX were separated from modern people by approximately 320 generations (8000 years ago), we calculated that C ≈ 2. Therefore, to convert IBD median lengths (Table 4) into the time for the last common ancestor, we used the formula ***L =* 2/(2 *× g × r***) for modern people and ***L =* 2*/(g × r***) for ancestral vs. modern genomes. These calculated ages of IBD separation are presented in the Table 5. Finally, we would like to stress that the years we calculated for last common ancestors for shared IBDs do not necessarily equate to the dates of separation for two populations. In some instances, a calculated time may reflect the date of admixture of two populations with another group of people that brought common IBD fragments with them. In other words, the last common ancestor for an IBD fragment between a pair of individuals should be distinguished from the last common ancestors between a pair of populations. Thus, the title of Table 5 is “Time of Last Common Ancestor or Admixture”. We verified that several of the calculated ages in Table 5 are congruent to the data in previous publications. For example, our estimations for the admixture of Neanderthals with ancestors of modern humans around 60–70 thousand years ago corresponds to previous estimations, and the separation of African and Oceania populations around 125 thousand years ago corresponds to a publication by Pagani and co-authors [4].

The multidimensional scaling (MDS) was made with the cmdscale() function from the stats R package [58]. The input data were given as the matrix which values are the numbers of shared IBD fragments between two individuals. The size of the matrix was 3121. Since the upper triangle of the matrix differed a little bit from the lower triangle because of peculiarities of computational method, we forced the matrix to be fully symmetrical by mirroring the lower triangle to upper triangle. Then we normalized the values as follows. The maximum value was found in the matrix and each value of the matrix was divided by this maximum value. The distance (dissimilarity) between individuals was estimated following the formula: d = (1 − s)/s according to Ignatov and co-authors 2012 [59], where s is a normalized number of shared IBD fragments between two individuals.

## Figures and Tables

**Table 1:** Average number of shared identical-by-descent (IBD) segments between populations.

Identifiers	Populations	Studied Populations from Americas															
		Cac	Cha	CLM	Col	Kar	May	Mix	MXL	PEL	Pia	Pim	PUR	Que	Sur	Wic	Zap
Cac_AMR	Cachi_Argentina	**53.5**	4.5	1.3	59.3	3.8	6.7	6.0	2.7	4.3	8.3	5.4	1.1	30.3	9.0	9.2	5.2
Cha_AMR	S_Chane:Argentina	5.3	**N/A**	0.8	9.1	13.7	12.3	11.8	2.8	4.8	24.8	6.8	0.6	9.0	19.8	80.8	10.6
CLM_AMR	Colombians_Medellin_Colombia	1.2	0.7	**25.6**	1.0	0.9	0.8	1.8	12.0	8.3	1.2	0.9	12.7	1.2	0.8	0.6	1.9
Col_AMR	Colla_Argentina	60.6	7.8	1.0	**86.1**	6.2	6.2	6.2	2.4	4.3	6.7	6.7	0.6	31.6	6.2	10.1	6.1
Kar_AMR	B_Karitiana:Brazil	4.4	14.2	1.1	6.2	**154.5**	9.5	8.2	2.5	4.3	16.3	4.9	0.5	7.3	66.9	6.7	7.1
May_AMR	S_Mayan:Mexico	7.1	11.5	1.0	6.6	9.8	**42.9**	19.4	4.9	5.9	13.2	17.2	0.9	11.8	11.2	5.9	20.9
Mix_AMR	B_Mixe:Mexico	6.5	12.4	1.9	6.5	8.3	20.2	**65.7**	6.2	5.2	10.9	17.2	1.5	8.7	7.9	6.7	41.1
MXL_AMR	Mexican_Ancestry_Los_Angeles	2.2	1.9	11.9	2.1	1.9	3.5	4.1	**17.6**	11.2	2.4	3.8	10.7	1.8	2.2	2.0	5.9
PEL_AMR	Peruvians_Lima_Peru	2.5	2.6	8.0	2.6	3.2	4.0	3.5	10.5	**18.3**	3.1	3.4	6.9	5.0	2.4	2.2	4.5
Pia_AMR	S_Piapoco:Colombia	9.2	25.6	1.4	6.7	16.0	12.8	10.9	3.4	5.4	**153.2**	7.8	0.5	12.4	20.6	5.3	9.9
Pim_AMR	S_Pima:Mexico	5.4	8.3	1.1	7.3	5.1	18.3	17.3	6.2	5.3	8.9	**168.9**	0.3	9.8	8.5	7.1	17.1
PUR_AMR	Puerto_Ricans_from_Puerto_Rico	1.1	0.4	12.8	0.6	0.4	0.8	1.4	10.9	7.1	0.5	0.3	**29.9**	0.8	0.5	0.4	1.2
Que_AMR	S_Quechua:Peru	32.3	9.6	1.3	33.4	6.9	12.0	8.1	2.5	9.1	11.9	8.6	0.9	**58.5**	9.1	5.9	9.2
Sur_AMR	S_Surui:Brazil	9.9	21.5	0.8	6.2	65.9	11.2	8.2	2.8	4.2	21.0	8.9	0.6	8.8	**153.2**	7.0	9.5
Wic_AMR	Wichi_Argentina	9.6	80.3	0.6	10.2	6.3	5.7	6.2	2.7	3.5	5.3	6.9	0.4	5.9	6.8	**236.9**	10.2
Zap_AMR	S_Zapotec:Mexico	5.4	9.0	2.2	6.1	7.1	20.9	39.6	8.0	6.4	10.3	16.0	1.2	9.0	9.1	10.2	**78.1**
Ale_ARC	S_Aleut:Russia	1.0	0.8	2.7	1.4	3.2	4.8	2.3	3.3	2.4	3.7	4.7	2.3	2.5	4.1	2.0	2.5
Chu_ARC	S_Chukchi:Russia	0.8	0.7	1.3	0.6	1.1	1.6	1.7	1.6	1.1	0.6	2.2	1.3	0.6	0.8	0.8	0.9
Esk_ARC	S_Eskimo_Sireniki:Russia	1.3	2.7	0.4	1.1	1.8	2.2	2.5	1.5	1.6	2.2	5.5	0.4	1.8	1.9	1.8	3.1
Ite_ARC	S_Itelman:Russia	0.3	2.9	0.6	0.0	1.4	2.1	1.3	1.5	1.3	0.7	2.6	0.6	1.4	2.1	0.0	4.1
Kor_ARC	Koryaks	0.3	0.4	0.1	0.3	0.2	0.6	0.4	0.3	0.3	0.4	0.8	0.1	0.4	0.2	0.5	0.3
Tli_ARC	S_Tlingit:Russia	1.5	0.8	4.4	1.7	0.5	2.6	2.6	4.2	3.2	1.5	3.5	4.2	2.6	1.5	0.8	1.1
Ulc_ARC	S_Ulchi:Russia	0.6	0.7	0.3	0.7	0.4	1.7	0.4	0.9	1.0	0.0	0.9	0.2	0.2	0.7	0.2	1.7
Alt_CAS	Altaian	0.1	0.2	1.3	0.5	0.4	0.6	0.4	1.3	1.0	0.1	0.4	1.1	0.2	0.1	0.1	0.4
Ish_CAS	Ishkasim_Tajikistan	0.4	0.0	2.9	0.0	0.0	0.4	0.4	2.2	1.0	0.0	0.0	2.6	0.3	0.0	0.2	0.4
Kal_CAS	Kalmyk	0.4	1.8	1.1	0.5	1.8	0.0	0.0	1.1	0.9	0.0	0.0	1.1	0.6	0.0	0.0	0.9
Kaz_CAS	Kazakhs_Kazakhstan	0.3	0.0	1.9	0.3	0.2	0.0	0.2	1.4	0.8	0.3	0.0	1.7	0.0	0.0	0.2	0.3
Kyr_CAS	S_Kyrgyz:Kyrgyzystan	0.3	0.0	1.4	0.2	0.3	0.3	0.4	1.4	1.0	0.2	0.4	1.5	0.4	0.1	0.1	0.7
Rus_CAS	Rushan-Vanch_Tajikistan	0.2	0.0	3.4	0.2	0.0	0.0	0.6	2.6	2.3	0.4	0.7	3.3	0.6	0.0	0.2	0.0
Shu_CAS	Shugnan_Tajikistan	1.1	0.0	3.4	0.0	0.0	0.0	0.3	3.2	1.6	0.0	0.0	3.5	0.0	0.0	0.0	0.0
Taj_CAS	Tajiks	0.1	0.5	4.2	0.5	0.0	0.5	0.6	3.9	2.0	0.0	0.2	4.0	0.3	0.3	0.0	0.7
TKm_CAS	Turkmens_Uzbekistan	0.1	0.0	2.4	0.2	0.0	0.6	0.4	2.4	1.4	0.0	0.3	2.4	0.0	0.3	0.2	0.8
Uyg_CAS	Uygurs_Kazakhstan	0.2	0.0	2.5	0.1	0.0	0.0	0.4	2.1	1.3	0.0	0.4	2.4	0.3	0.2	0.1	0.5
Uzb_CAS	Uzbek	0.2	0.6	2.7	0.1	0.2	0.5	0.5	2.5	1.5	0.0	0.0	2.6	0.7	0.3	0.9	0.0
Yag_CAS	Yaghnobi_Tajikistan	0.0	0.0	3.1	1.3	0.0	1.6	0.9	3.0	1.8	0.0	0.0	3.7	0.5	0.0	0.0	0.0

This table represents an illustrative fragment of the complete version of 243 × 243 Supplementary Table S1. The number in the crossing for a row, presenting a population ***A,*** and a column, presenting population ***B,*** shows the average number of shared IBD fragments for a pair of individuals in which one person belongs to population ***A*** while the other person from population ***B***. In a vast majority of cases, when ***A*** and ***B*** represent the same population, the number of shared IBD fragments between individuals of this population is the highest compared to the number of shared IBD fragments between this population and another one. In rare cases, a population in our study is represented by a sole individual (e.g., Chane from Argentina). Because the number of shared IBD fragments for the same person does not make biological sense, the table shows **N/A** value for intra-population data for single individual.

**Table 2:** Example of ranking data for numbers of shared IBD fragments for Russian population (identifier: *Rus_EUR*).

#IBDs	Pop ID	Population Name
27.29	Vep_EUR	Vepsas_Russia
23.92	Kar_EUR	Karelians
23.39	Rus_EUR	S_Russian:Russia
23.11	Est_EUR	S_Estonian:Estonia
23.00	Lat_EUR	Latvians
22.86	Fin_EUR	S_Finnish:Finland
22.37	Ing_EUR	Ingrians_Russia_North
21.43	Kom_EUR	Komis_Russia
21.15	Lit_EUR	Lithuanians
20.04	FIN_EUR	Finnish_in_Finland
19.58	Bel_EUR	Belarusians
19.37	Pol_EUR	S_Polish:Poland
19.36	Saa_EUR	S_Saami:Finland
18.91	Ukr_EUR	Ukrainians
18.20	Tli_ARC	S_Tlingit:Russia
17.81	Cos_EUR	Cossacks_Ukraine
17.79	Mor_EUR	Mordvins_Russia
17.08	Nor_EUR	S_Norwegian:Norway
16.03	Hun_EUR	S_Hungarian:Hungary
15.88	Kry_EUR	Kryashen-Tatars_Russia
15.19	Swe_EUR	Swedes_Sweden
…	…	…
5.52	Tur_MDE	S_Turkish:Turkey
5.42	NOc_CAU	S_North_Ossetian:Russia
5.30	Chu_ARC	S_Chukchi:Russia
5.20	Haz_SAS	S_Hazara:Pakistan
5.15	Che_CAU	S_Chechen:Russia
5.03	Cir_CAU	Circassians_Russia_Caucaz
4.92	Kum_CAU	Kumyks_Russia
4.92	Sar_EUR	B_Sardinian:Italy
4.79	CLM_AMR	Colombians_Colombia
4.78	Sel_SIB	Selkups_Russia
4.73	Rom_EUR	Roma_Bosnia-Herzegovina
4.71	Tub_SIB	S_Tubalar:Russia
4.64	Taj_CAS	Tajiks
…	…	…
0.03	Dus_OCE	S_Dusun:Brunei
0.03	Igo_OCE	S_Igorot:Philippines
0.01	Luh_AFR	S_Luhya:Kenya
0	Con_AFR	Congo-pygmies_
0	Esa_AFR	S_Esan:Nigeria
0	Cha_AMR	S_Chane:Argentina
0	Ami_EAS	S_Ami:Taiwan
0	Haw_OCE	S_Hawaiian:USA
0	Gon_SAS	Gond_India

The first column shows the average numbers of shared IBD fragments (per pair of individuals) between Russian population with others. The second column shows population identifier, and the third—population name. Table 2 shows the first 21 rows with the highest numbers of IBDs, 12 rows from the middle of this table and 9 last rows with the least numbers of IBDs. The entire set of ranking tables for all populations are presented in the Supplementary File S1.

**Table 3:** Nine Distinct Human Genetics Regions (DHGR), each characterized by three reference populations that share the least number of IBD fragments with the rest of the world.

*IDs*	Unique Genetic Regions	3 Reference Populations
AFE	East Africa	Luhya Webuye Kenya (LWK); Dinka (Sudan); Masai (Kenya)
AFW	West Africa	Yoruba (YRI); Esan Nigeria (ESN); Mende Sierra Leone (MSL)
AMR	America	Piapoco (Columbia); Wichi (Argentina); Karitiana (Brazil)
ARC	Arctica (N Eurasia + N. America)	Koryaks (Russia); Eskimo Sireniki (Russia); Chukchi (Russia)
EAS	East Asia	Han China South (CHS); Japanese Tokyo (JPT); Miao (China)
EUR	North Europe	Swedes (Sweden); Estonians (Estonia); Germans (Germany)
SAS	Hindustan Peninsula	Sri Lankans from UK (STU); Gujarati from Texas (GIH); Mala (India)
OCE	Oceania + Australia	Australians (Australia); Papuan (Papua New Guinea); Agta (Philippines)
MDE	Middle East	Arabs (Israel); Saudi-Arabians; Palestinian (Israel Central)

**Table 4:** Median lengths of shared IBD fragments between populations from major geographical regions (in 1000 nucleotides).

	AFR	AMR	ARC	CAS	CAU	EAS	EUR	MDE	OCE	SAS	SIB	NEA	ANC
**AFR**	157	128	100	52	96	29	117	108	17	47	54	22	200
**AMR**	128	635	408	322	415	330	435	315	52	316	334	80	N/A
**ARC**	100	408	1253	536	475	398	609	409	180	384	683	60	531
**CAS**	52	322	536	641	492	504	522	422	288	457	668	62	412
**CAU**	96	415	475	492	704	428	491	469	44	404	518	57	487
**EAS**	29	330	398	504	428	558	395	354	39	392	466	63	N/A
**EUR**	117	435	609	522	491	395	670	443	61	453	580	58	585
**MDE**	108	315	409	422	469	354	443	473	25	372	407	57	465
**OCE**	17	52	180	288	44	39	61	25	482	54	254	77	N/A
**SAS**	47	316	384	457	404	392	453	372	54	478	440	63	403
**SIB**	54	334	683	668	518	466	580	407	254	440	996	62	692
**NEA**	22	80	60	62	57	63	58	57	77	63	62	N/A	N/A
**ANC**	200	N/A	531	412	487	N/A	585	465	N/A	403	692	N/A	N/A

**Table 5:** Time (in 1000 years) for last common ancestors of shared IBD fragments between populations from major geographical regions.

	AFR	AMR	ARC	CAS	CAU	EAS	EUR	MDE	OCE	SAS	SIB	NEA	ANC
**AFR**	13.5	16.6	21.2	40.7	22.1	73.1	18.1	19.6	124	45.1	39.2	192	21.2
**AMR**	16.6	3.3	5.2	6.6	5.1	6.4	4.9	6.7	40.7	6.7	6.3	53.0	N/A
**ARC**	21.2	5.2	1.7	4.0	4.5	5.3	3.5	5.2	11.8	5.5	3.1	70.6	8.0
**CAS**	40.7	6.6	4.0	3.3	4.3	4.2	4.1	5.0	7.4	4.6	3.2	68.3	10.3
**CAU**	22.1	5.1	4.5	4.3	3.0	5.0	4.3	4.5	48.2	5.2	4.1	74.3	8.7
**EAS**	73.1	6.4	5.3	4.2	5.0	3.8	5.4	6.0	54.3	5.4	4.5	67.3	N/A
**EUR**	18.1	4.9	3.5	4.1	4.3	5.4	3.2	4.8	34.7	4.7	3.7	73.1	7.2
**MDE**	19.6	6.7	5.2	5.0	4.5	6.0	4.8	4.5	84.7	5.7	5.2	74.3	9.1
**OCE**	124	40.7	11.8	7.4	48.2	54.3	34.7	84.7	4.4	39.2	8.3	55.0	N/A
**SAS**	45.1	6.7	5.5	4.6	5.2	5.4	4.7	5.7	39.2	4.4	4.8	67.3	10.5
**SIB**	39.2	6.3	3.1	3.2	4.1	4.5	3.7	5.2	8.3	4.8	2.1	68.3	6.1
**NEA**	192	53.0	70.6	68.3	74.3	67.3	73.1	74.3	55.0	67.3	68.3	N/A	N/A
**ANC**	21.2	N/A	8.0	10.3	8.7	N/A	7.2	9.1	N/A	10.5	6.1	N/A	N/A

**Table 6:** Characteristics of ancient DNA samples from prehistoric people.

Sample	Species	Site/Region	Dating (YA)	Coverage	Reference
Altai, Denisova5	*H. neanderthalensis*	Denisova Cave, Altai mountains	52,000 (122,000) *	50	[53]
Chagyrskaya 8	*H. neanderthalensis*	Chagyrskaya Cave Altai mountains	60,000 (80,000) *	27	[54]
Vindija 33.19	*H. neanderthalensis*	Vindija Cave, northern Croatia	45,000 (52,000) *	30	[55]
Ustishim	*H. sapiens*	Settlement of Ust’-Ishim in western Siberia	45,000	40	[56]
Denisova	*H. denisova*	Denisova Cave, Altai mountains	40,000 (72,000) *	30	[18]
Stuttgart	*H. sapiens*	Germany	7000	19	[24]
Loschbour	*H. sapiens*	Loschbour rock shelter, Luxembourg	8000	22	[24]

* In brackets the dates inferred using the DNA-based method are presented [55].

## Supplementary Materials

The following are available online at https://www.mdpi.com/2079-7737/9/11/392/s1: Table S1: Average numbers and lengths of shared IBD segments between populations. In this Microsoft Excel table in Sheet-1 (IBD-Number) the number in the crossing for a row, presenting a population A, and a column, presenting population B, shows the average number of shared IBD fragments for a pair of individuals in which one person belongs to population A while the other person from population B. In Sheet-2 (IBD-Normalized-number) cells show the normalized numbers of shared IBD fragments for a pair of individuals (see Materials and Methods). In Sheet-3 (IBD-Median-length) cells show the median length (in Kb) of shared IBD fragments for a pair of individuals. In Sheet-4 (IBD-Mean-length) cells show the mean length (in Kb) of shared IBD fragments for a pair of individuals. Table S2: Description of individuals and populations. Sheet-1 (Studied Individuals) presents the description of studied individuals by public projects. Sheet-2 (IDs for 3121 individuals) presents the list of all 3121 individuals in the alphabetical order. Sheet-3 (List of Populations) presents the names of all studied populations. Table S3: The complete table of numbers of shared IBD fragments for every pair of studied individuals. Table S4: Relative genetic relatedness of studied populations to the nine Distinct Human Genetics Regions shown in percentages. Figure S1: The results of MDS applied to dissimilarity matrix on 3121 individuals. Supplementary File S1. This is an archived and compressed extra-large file, Khvorych_SupplementarySF1.tar.gz, which size is 6.2 GB. It is available from our web page (http://bpg.utoledo.edu/~afedorov/lab/ATLAS2.html). The file presents the following databases of very rare SNP alleles and large datasets described in the paper: vrGV_1KG_A2_$chr (22 files for each chromosome); NONvrGV_A2_$chr (22 files for each chromosome); NONvrGVposition_A2_$chr (22 files for each chromosome); vrGVdb_1K_S_E_V_A7_all_v3 (main database of very rare SNP alleles); directory: INDIVIDUAL_vrGVdbs_v3 (contains 3121 files); directory DATAjune2020 (contains 6242 files dat3 and dat4); directory: RANKjune10 (contains 244 files); Supplementary File S2. This is an archived file that contains all programs, supplementary data files, Instruction Manual, and electronic Lab protocol with all command lines and comments for described computations.
